# Mast cell heterogeneity underlies different manifestations of food allergy in mice

**DOI:** 10.1371/journal.pone.0190453

**Published:** 2018-01-25

**Authors:** Sara Benedé, M. Cecilia Berin

**Affiliations:** Jaffe Food Allergy Institute, Icahn School of Medicine at Mount Sinai, New York, NY, United States of America; Harvard Medical School, UNITED STATES

## Abstract

Food can trigger a diverse array of symptoms in food allergic individuals from isolated local symptoms affecting skin or gut to multi-system severe reactions (systemic anaphylaxis). Although we know that gastrointestinal and systemic manifestations of food allergy are mediated by tissue mast cells (MCs), it is not clear why allergen exposure by the oral route can result in such distinct clinical manifestations. Our aim was to assess the contribution of mast cell subsets to different manifestations of food allergy. We used two common models of IgE-mediated food allergy, one resulting in systemic anaphylaxis and the other resulting in acute gastrointestinal symptoms, to study the immune basis of allergic reactions. We used responders and non-responders in each model system, as well as naïve controls to identify the association of mast cell activation with clinical reactivity rather than sensitization. Systemic anaphylaxis was uniquely associated with activation of connective tissue mast cells (identified by release of mouse mast cell protease (MMCP) -7 into the serum) and release of histamine, while activation of mucosal mast cells (identified by release of MMCP-1 in the serum) did not correlate with symptoms. Gastrointestinal manifestations of food allergy were associated with an increase of MMCP-1-expressing mast cells in the intestine, and evidence of both mucosal and connective tissue mast cell activation. The data presented in this paper demonstrates that mast cell heterogeneity is an important contributor to manifestations of food allergy, and identifies the connective tissue mast cell subset as key in the development of severe systemic anaphylaxis.

## Introduction

Mast cells (MCs) are the main effector cells in IgE-mediated food allergy [1], but the understanding of their role is incomplete. During the allergic response, food allergen cross-links IgE bound to MCs via FcεRI, resulting in activation and release of preformed granule contents, rapidly synthesized lipid mediators or cytokines and chemokines. Symptoms triggered by foods occur within 2 hours of ingestion, and can affect single or multiple organ systems, including the skin (hives), the respiratory tract (bronchoconstriction, cough), and the gastrointestinal tract (nausea, vomiting, diarrhea) [2]. Anaphylaxis refers to serious generalized symptoms that affect the respiratory tract or cardiovascular system in addition to skin or mucosal symptoms [3]. Of the vast number of bioactive molecules that are produced by MCs, histamine and platelet activating factor (PAF) contribute to peanut-induced systemic anaphylaxis in mice and humans [4–6], while serotonin and PAF are the main mediators that contribute to gastrointestinal symptoms in mice [7].

Mast cells are derived from hematopoietic stem cells, which give rise to mast cell progenitors that circulate in the blood and enter the tissues, where they undergo differentiation and maturation to become mature MCs. The different microenvironments found in tissues modulate the morphology and features of MCs and therefore specific subpopulations are observed in distinct tissues [8]. Mouse MCs are classified based on their anatomic location in two groups, mucosal (MMCs) and connective tissue (CTMCs) mast cells [9]. In humans, tissue distribution is not as clearly demarcated as in rodents [10]. Most human tissues have a mixed population of mast cell types that are distinguished by their protease composition. Tryptase-only MCs, are located predominantly in the alveolar wall and gastric mucosa similar to MMCs in rodents. Chymase-only MCs, or both tryptase- and chymase-positive MCs are located predominantly in the skin and intestinal submucosa like CTMCs in rodents. For all subsets, recent evidence suggests that the expression of their secretory granule proteases is directed by the local tissue in which the cells reside [11]. It is also known that they differ in their amine content as well as in some of their functional properties [9], but the biological implications of these differences are still poorly understood and their roles in the context of food allergy, remain unexplored. Our goal was to examine the role of tissue-specific subsets of MCs in food allergy, including models with systemic or gastrointestinal manifestations of food allergy.

## Material and methods

### Antigens and adjuvants

Ovalbumin (OVA, grade V) was purchased from Sigma-Aldrich (St. Louis, MO). Staphylococcal enterotoxin B (SEB) was from Toxin Technologies (Sarasota, FL).

### Mice

BALB/c and C3H/HeJ mice were obtained from Charles River Laboratories (Wilmington, MA). All animal procedures were approved by the Institutional Animal Care and Use Committee of the Icahn School of Medicine at Mount Sinai (Protocol LA11-00273). Mice were euthanized by cervical dislocation while under anesthesia. Mice were anesthetized with isofluorane (inhalation) for immediate euthanasia and for sensitization or ketamine and xylazine (intraperitoneal) for blood sampling. Mice were routinely monitored for signs of illness, injury, or behavioral changes by persons trained to recognize such signs. In the case of illness or injury leading to distress, and under guidance from the veterinary staff at the Center for Comparative Medicine and Surgery, mice were removed from the experiment and euthanized. Allergen challenge induces acute reactions that resolve within an hour, no additional medications were provided to treat reactions. In the case of injury or severe symptoms (for example seizures) following allergen challenge, mice were immediately euthanized (4 of 81 C3H/HeJ mice).

### Epicutaneous sensitization of mice

**Fig 1:**
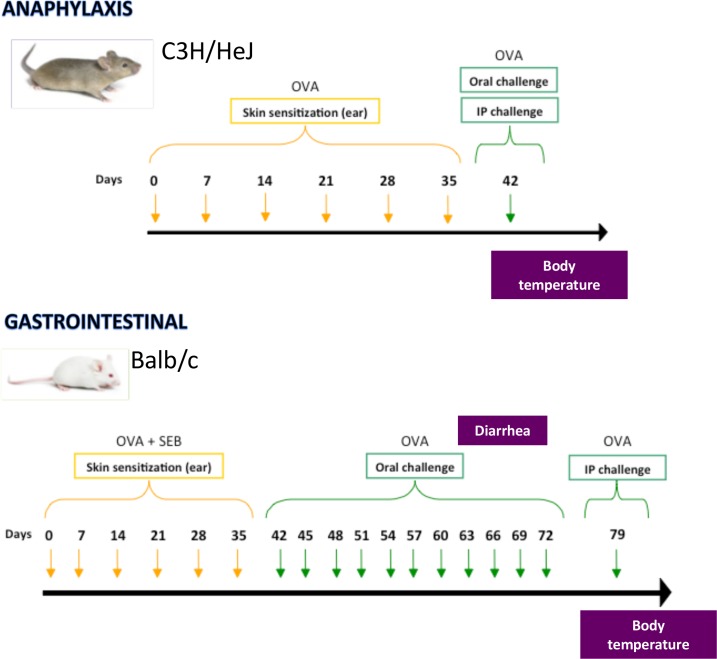
**Mouse models of food allergy:** Protocols of sensitization to OVA and induction of anaphylaxis in C3H/HeJ mice (A) and allergic diarrhea in Balb/c mice (B).

4–5 week old mice were anesthetized and exposed to 100 μg of ovalbumin (OVA, grade V) in 50 μl of PBS spread thinly on the ear pinnae to dry. SEB was applied with OVA at a dose of 10 μg in Balb/c mice. Mice were exposed weekly for a total of 6 weeks, and challenged a minimum of one week after the last exposure (Fig 1).

### Allergen challenge and assessment of symptoms

Mice underwent an oral or intraperitoneal challenge to OVA (grade V) (50 mg or 50 μg, respectively). Body temperature was measured before and 30 minutes after challenge using a rectal thermometer (WPI Instruments, Sarasota, FL). Anaphylaxis severity was measured by drop in body temperature and gastrointestinal symptoms (diarrhea) were marked as present or absent.

### Assessment of antigen-specific immunoglobulins

Blood samples were obtained 30 minutes after allergen challenge. OVA-specific IgE was measured by capture ELISA using DIG-labeled OVA as detection [12]. OVA-specific IgG1, and IgG2a were measured by ELISA using biotinylated monoclonal detection antibodies (all from BD Biosciences, San Diego, CA) [13].

### Immunostaining

MMCP-1 and MMCP-7 were detected in fresh frozen and formalin-fixed paraffin embedded tissues, respectively. Immunohistochemical detection of MMCP-1 was performed using a mAb from eBioscience at a final concentration of 10 μg/mL followed by biotinylated anti-rat IgG (Vector laboratories). Biotinylated anti-mtryptase B-1/MCPT-7 at 0.5 μg/mL (R&D Systems, Minneapolis, MN) was used to detect MMCP-7. Ab binding was detected by ABC reagent (Vector laboratories, Burlingame, CA), and DAB chromogen (Vector Laboratories).

### Mast cell staining

Mast cells were identified by chloroacetate esterase staining of paraffin sections of formalin-fixed mouse jejunum [14]. Mast cells per 10 high-power fields were counted and averaged per mouse.

### Skin, intestine, lung and peritoneal cavity RNA isolation

Ears, jejunum and lung were harvested and peritoneal lavage was performed from naïve or OVA-sensitized mice. In Balb/c mice, jejunum was harvested after repeated OVA challenge. Total RNA was isolated with Trizol (Invitrogen, Life Technologies, Grand Island, NY, USA), followed by RNA clean up with RNeasy mini kit (Qiagen, Valencia, CA).

### Real-time PCR

RT-PCR was performed starting from 1 μg total RNA, using SuperScript II reverse transcriptase (Invitrogen). cDNA was amplified using the Power SYBR Green PCR Master Mix (Applied Biosystems, Life Technologies) and run on CFX384 Touch™ real-time PCR detection system (Bio Rad, Hercules, CA), using the primers described in Table 1. Beta-actin was used as the housekeeping gene. Relative quantification was performed using the comparative threshold cycle method (2^ΔCt^). All amplifications were carried out in triplicates.

**Table 1:** List of primers for RT-PCR.

Gene	Forward sequence 5’-3’	Reverse primer 5’-3’
MMCP-1	CAGATGTGGTGGGTTTCTCA	GCTCACATCATGAGCTCCAA
MMCP-2	AGGCCCTACTATTCCTGATGG	ATGTAAGGACGGGAGTGTGG
MMCP-4	GCTACCTGTGGTGGGTTTCT	TCACATCATGAGCTCCAAGG
MMCP-5	CAGGCCCTGGATCAATAAGA	GGCACACAAAACCTGCACTA
MMCP-6	CTATCCAGGGTCAGGCAAGA	GACAGGGGAGACAGAGGACA
MMCP-7	GACCCCAACAAGGTCAGAGT	TGTAGAAGTCGGGGTGTGTG
nMC-CPA	TCCAGGAACCAAAACTCCAC	CATTGAGGCATGGTTTGTG

### Enzyme immunoassays

MMCP-1 was quantified in mouse serum by ELISA kit (eBioscience, San Diego, CA) and MMCP-7 using anti-mouse tryptase B-1/MCPT-7, biotinylated anti- mouse tryptase B-1/MCPT-7 and recombinant mouse tryptase B-1 (R&D Systems, Minneapolis, MN). Histamine and serotonin were quantitated in mice serum by using immunoassay EIA kits (Beckman coulter, Jersey City, NY) and leukotrienes using C4/D4/E4 EIA Kit (Oxford Biomedical Research, Oxford, MI).

## Results

### Tissue mast cell protease profiles in food allergy mouse models

We hypothesized that different MC subsets would underlie gastrointestinal versus anaphylactic symptoms of food allergy. We used modifications of two well-characterized models of food allergy (Fig 1). The first, in Balb/c mice, results in gastrointestinal symptoms (diarrhea) but a lack of systemic anaphylaxis when mice are orally challenged [7, 15]. The second, in C3H/HeJ mice, results in systemic anaphylaxis without gastrointestinal symptoms upon oral challenge [13, 16]. Both models use the egg allergen ovalbumin, egg being the second most common food allergy of childhood after milk. Although the allergic diarrhea model was first reported as initiated by systemic (alum) immunization, we used epicutaneous exposure to OVA to initiate both models. Although systemic anaphylaxis together with gastrointestinal symptoms has been reported in Balb/c mice after oral challenge [17, 18], we did not record any drop in body temperature in epicutaneously sensitized Balb/c mice with diarrhea symptoms (not shown).

**Fig 2:**
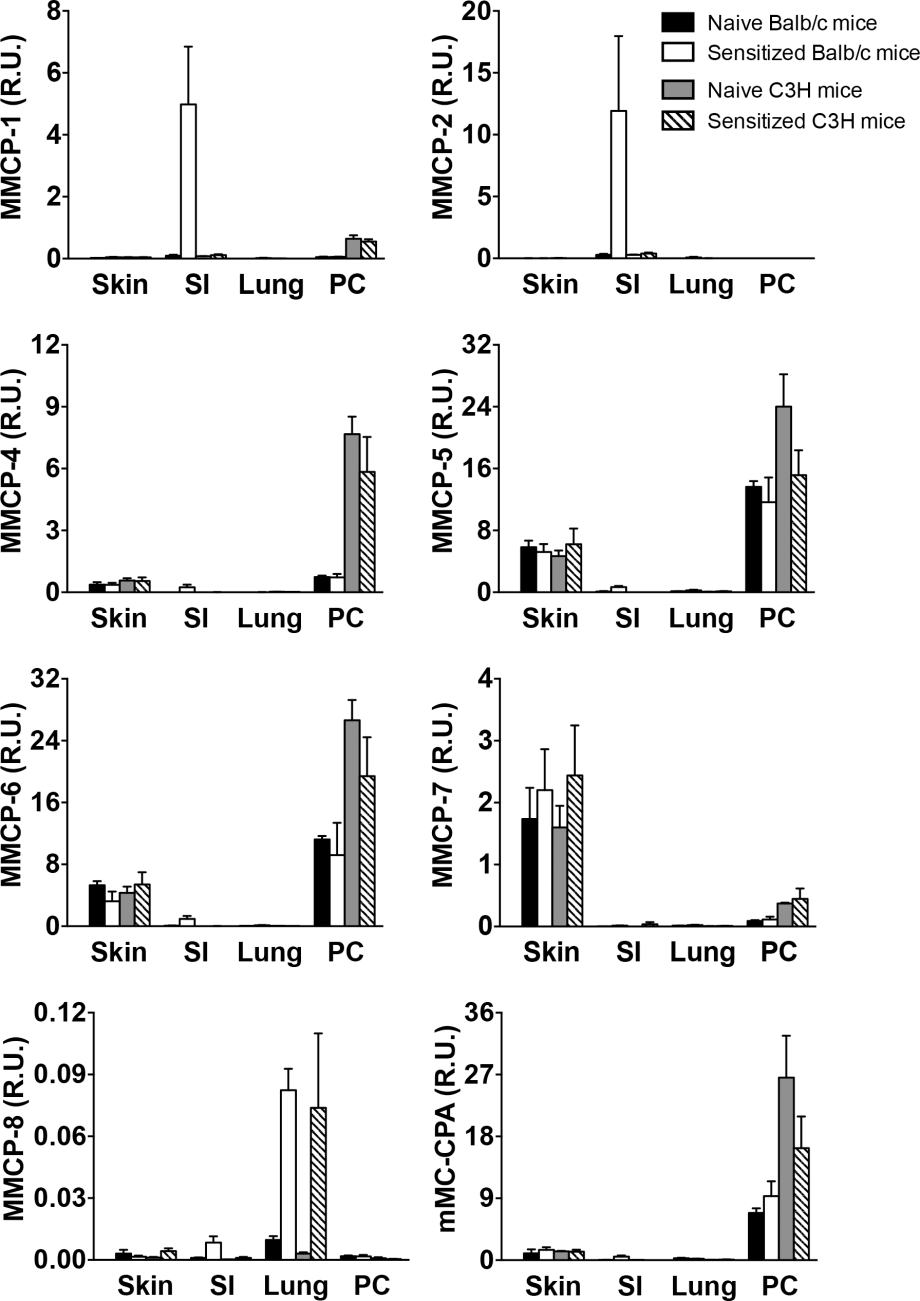
Mast cell protease expression from different tissues. Real-time RT-PCR for MMCP-1, MMCP-2, MMCP-4, MMCP-5, MMCP-6, MMCP-7, MMCP-8 and mMC-CPA in skin, small intestine (SI), lung, and peritoneal cavity (PC) from naïve and sensitized C3H/HeJ and Balb/c mice. Balb/c mice were previously challenged to OVA to prime mice for symptoms. Relative expression refers to data normalized to the housekeeping gene. Data are mean ± SEM of 4 different experiments.

**Fig 3:**
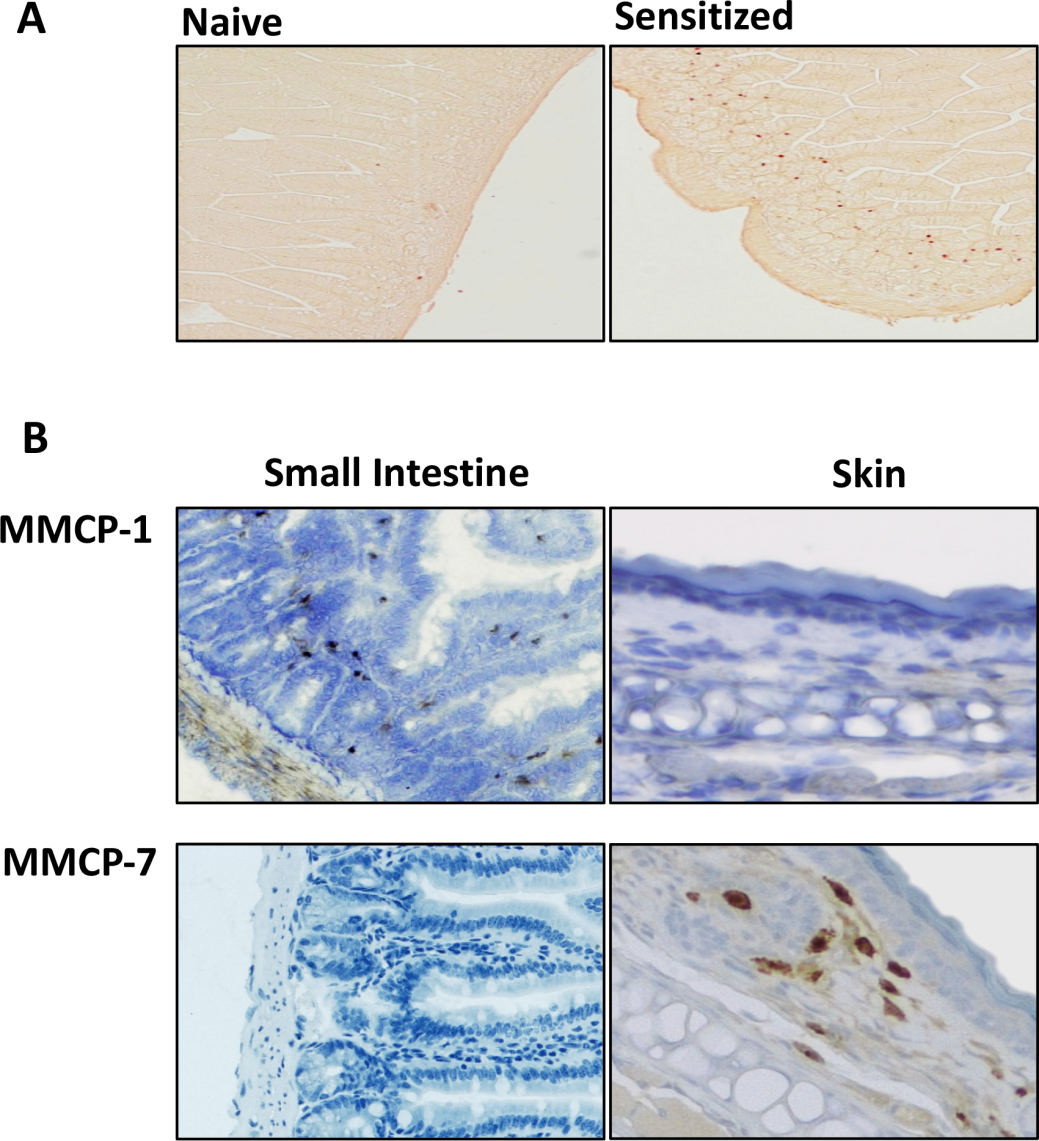
Tissue mast cells in skin and jejunum. (A) Jejunum from naïve or sensitized and primed Balb/c mice was stained with chloroacetate esterase (red color) to identify mast cells. (B) Jejunum (intestine) from sensitized and primed Balb/c mice or skin (ear) was stained with antibodies to MMCP-1 and MMCP7 (dark brown color). Images are representative of the results of analysis of at least four mice per group.

Mast cells from different tissues express different proteases, and to verify this in our model systems, we analyzed MC protease mRNA expression in skin, small intestine (SI), peritoneal cavity and lung from naïve and sensitized C3H/HeJ and Balb/c mice (Fig 2). Balb/c mice had been repeatedly orally challenged to prime them for symptoms prior to tissue harvest. In the SI, we found a predominant expression of MMCP-1 and 2, while in skin, the MC proteases MMCP-4, 5, 6, 7 and carboxypeptidase dominated. In cells from the peritoneal cavity, the protease profile was similar to CTMCs from the skin but lacking expression of MMCP-7. Epicutaneously sensitized and orally challenged Balb/c mice, but not C3H/HeJ mice, demonstrated an increase in MMCP-1 and MMCP-2 expression in the SI (Fig 2), reflecting expansion of gastrointestinal MCs (Fig 3). Naïve and sensitized mice showed similar relative expression among the proteases, indicating that the protease content in MCs is not modified during the sensitization process. We also examined the expression of MMCP-8, a marker of basophils rather than MCs [19]. MMCP-8 expression was restricted to the lungs, and interestingly was upregulated in both sensitized C3H/HeJ and Balb/c mice. We confirmed the tissue restriction of MMCP-1 and MMCP-7 by immunostaining (Fig 3). MMCP-1 was expressed in the sensitized small intestine, but not the skin, while MMCP-7 was expressed in the skin, but not the sensitized small intestine. For this reason, MMCP-1 and MMCP-7 were selected as markers of MMCs and CTMCs respectively. Although MMCP-2 showed intestinal tissue restriction (Fig 2), lack of commercial antibodies restricted its use as a marker of MMCs.

### Contribution of mast cell subsets to systemic manifestations of food allergy

**Fig 4:**
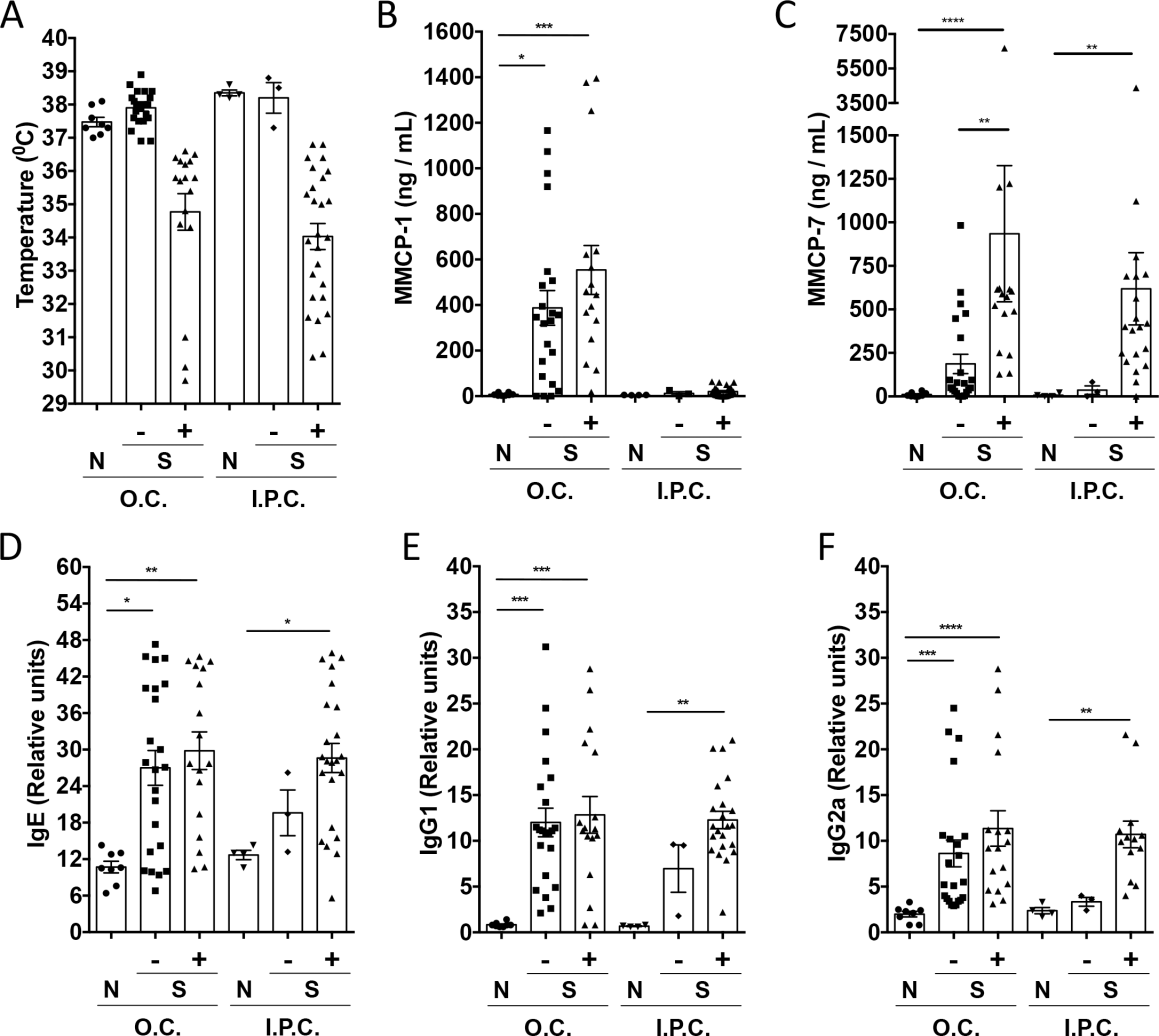
Contribution of mast cell subsets to food-induced anaphylaxis. C3H/HeJ mice (n = 40) were sensitized and orally (O.C.) or intraperitoneally (I.P.C.) challenged with OVA to induce anaphylaxis. Controls (N) included naïve mice that were not sensitized but challenged with OVA. Body temperature (A), MMCP-1 (B) MMCP-7 (C), OVA-specific IgE (D), OVA-specific IgG1 (E) and OVA-specific IgG2a (F) serum levels of mice measured 30 minutes after O.C. or I.P.C. in naïve (N) and symptomatic (+) or asymptomatic (-) sensitized (S) mice. Horizontal bars represent the mean value; error bars represent SEM. *p<0.05, ** p < 0.01, ***p<0.001, ***p<0.0001.

Oral challenge of sensitized C3H/HeJ mice resulted in systemic anaphylaxis in a population of responders (n = 17/40 total sensitized mice), defined by drop in body temperature (Fig 4A). OVA-specific IgE, IgG1 and IgG2a levels (Fig 4D–4F) measured in mouse sera after oral challenge with OVA did not discriminate responders from non-responders within the sensitized group, but were significantly elevated compared to naïve controls. Similarly, oral challenge with OVA resulted in a significant elevation in serum MMCP-1 in sensitized mice (Fig 4B, Table 2), but surprisingly these levels did not discriminate between responders and non-responders. MMCP-7 levels were significantly higher in those with systemic anaphylaxis than in sensitized and challenged mice without symptoms (Fig 4C, Table 2). Histamine serum levels were slightly increased in sensitized mice compared to naive controls and no differences were found between groups in serotonin and leukotriene (data not shown) levels.

**Table 2:** Adjusted p values comparing MMCP-1 and MMCP-7 levels between naïve, non-responder (NR) and responder (R) C3H mice after oral or intraperitoneal challenge.

	Oral		Intraperitoneal	
	MMCP1	MMCP7	MMCP1	MMCP7
**Naïve vs NR**	0.0102	0.0895	> 0.9999	> 0.9999
**Naïve vs R**	0.0002	< 0.0001	0.6016	0.0069
**NR vs R**	0.3789	0.0012	> 0.9999	0.0608

Systemic (intraperitoneal) challenge of sensitized C3H mice with OVA resulted in anaphylaxis in most mice, and responders showed elevated levels of OVA-specific IgE (Fig 4D), IgG1 (Fig 4E), IgG2a (Fig 4F), MMCP-7 (Fig 4C) and histamine levels (not shown). Responders did not differ from non-responders or naïve controls in levels of MMCP-1 (Fig 4B), serotonin and leukotrienes (not shown).

Correlations were calculated using the Spearman`s coefficient for each analyzed parameter after oral (Table 3) or intraperitoneal (Table 4) challenge. With drop in body temperature as a continuous variable of anaphylaxis severity, we observed significant correlation between temperature and MMCP-7, but not MMCP-1. We also observed that plasma histamine levels were significantly correlated with anaphylaxis severity and MMCP-7, but not MMCP-1, in response to either oral or systemic allergen challenge. These data suggest a critical role for activation of CTMCs, but not MMCs, in systemic anaphylaxis.

**Table 3:** Correlation between mast cell mediators measured in serum and anaphylaxis symptoms in C3H/HeJ mice after oral challenge.

Oral challenge: C3H/HeJ mice (Anaphylaxis)								
	Drop T	MMCP-1	MMCP-7	HISTAMINE	SEROTONIN	Leukotrienes	IgE	IgG1
**MMCP-1**	**0.101**	** **	** **	** **	** **	** **	** **	** **
	(0.545)							
**MMCP-7**	**0.620**	**0.428**	** **	** **	** **	** **	** **	** **
	(0.000)	(0.009)						
**HISTAMINE**	**0.472**	**0.267**	**0.791**	** **	** **	** **	** **	** **
	(0.006)	(0.139)	(0.000)					
**SEROTONIN**	**0.066**	**-0.132**	**0.264**	**-0.100**	** **	** **	** **	** **
	(0.813)	(0.645)	(0.380)	(0.810)				
**Leukotrienes**	**0.590**	**0.390**	**-0.366**	**0.111**	**-0.786**	** **	** **	** **
	(0.101)	(0.306)	(0.345)	(0.840)	(0.048)			
**IgE**	**0.068**	**-0.154**	**0.369**	**0.330**	**0.282**	**-0.763**	** **	** **
	(0.678)	(0.357)	(0.023)	(0.061)	(0.305)	(0.017)		
**IgG1**	**0.026**	**-0.233**	**0.054**	**0.068**	**-0.070**	**-0.415**	**0.766**	** **
	(0.873)	(0.165)	(0.753)	(0.712)	(0.804)	(0.288)	(0.000)	
**IgG2a**	**0.122**	**-0.091**	**0.204**	**0.254**	**0.326**	**-0.220**	**0.776**	**0.817**
	(0.461)	(0.592)	(0.227)	(0.160)	(0.253)	(0.567)	(0.000)	(0.000)

Bolded values indicate spearman correlation coefficient, values in brackets indicate p value. Drop T = drop in body temperature.

**Table 4:** Correlation between mast cell mediators measured in serum and anaphylaxis symptoms in C3H/HeJ mice after intraperitoneal challenge.

Intraperitoneal challenge C3H/HeJ (Anaphylaxis)								
	Drop T	MMCP-1	MMCP-7	HISTAMINE	SEROTONIN	Leukotrienes	IgE	IgG1
**MMCP-1**	**0.236**							
	(0.218)							
**MMCP-7**	**0.822**	**0.538**						
	(0.000)	(0.008)						
**HISTAMINE**	**0.620**	**0.308**	**0.632**					
	(0.005)	(0.199)	(0.024)					
**SEROTONIN**	**-0.075**	**-0.438**	**-0.017**	**-0.192**				
	(0.773)	(0.102)	(0.982)	(0.504)				
**Leukotrienes**	**0.316**	**0.632**	**0.105**	**-0.949**	**0.211**			
	(0.667)	(0.500)	(1.000)	(0.000)	(0.833)			
**IgE**	**0.413**	**0.147**	**0.215**	**-0.192**	**-0.130**	**0.949**		
	(0.036)	(0.475)	(0.325)	(0.469)	(0.678)	(0.167)		
**IgG1**	**0.394**	**-0.044**	**0.179**	**0.116**	**-0.453**	**0.316**	**0.517**	
	(0.051)	(0.833)	(0.414)	(0.679)	(0.159)	(0.667)	(0.008)	
**IgG2a**	**0.651**	**0.184**	**0.346**	**0.361**	**-0.348**	**-0.211**	**0.691**	**0.558**
	(0.000)	(0.378)	(0.106)	(0.185)	(0.281)	(0.667)	(0.000)	(0.004)

Bolded values indicate spearman correlation coefficient, values in brackets indicate p value. Drop T = drop in body temperature.

### Contribution of mast cell subsets to gastrointestinal manifestations of food allergy

**Fig 5:**
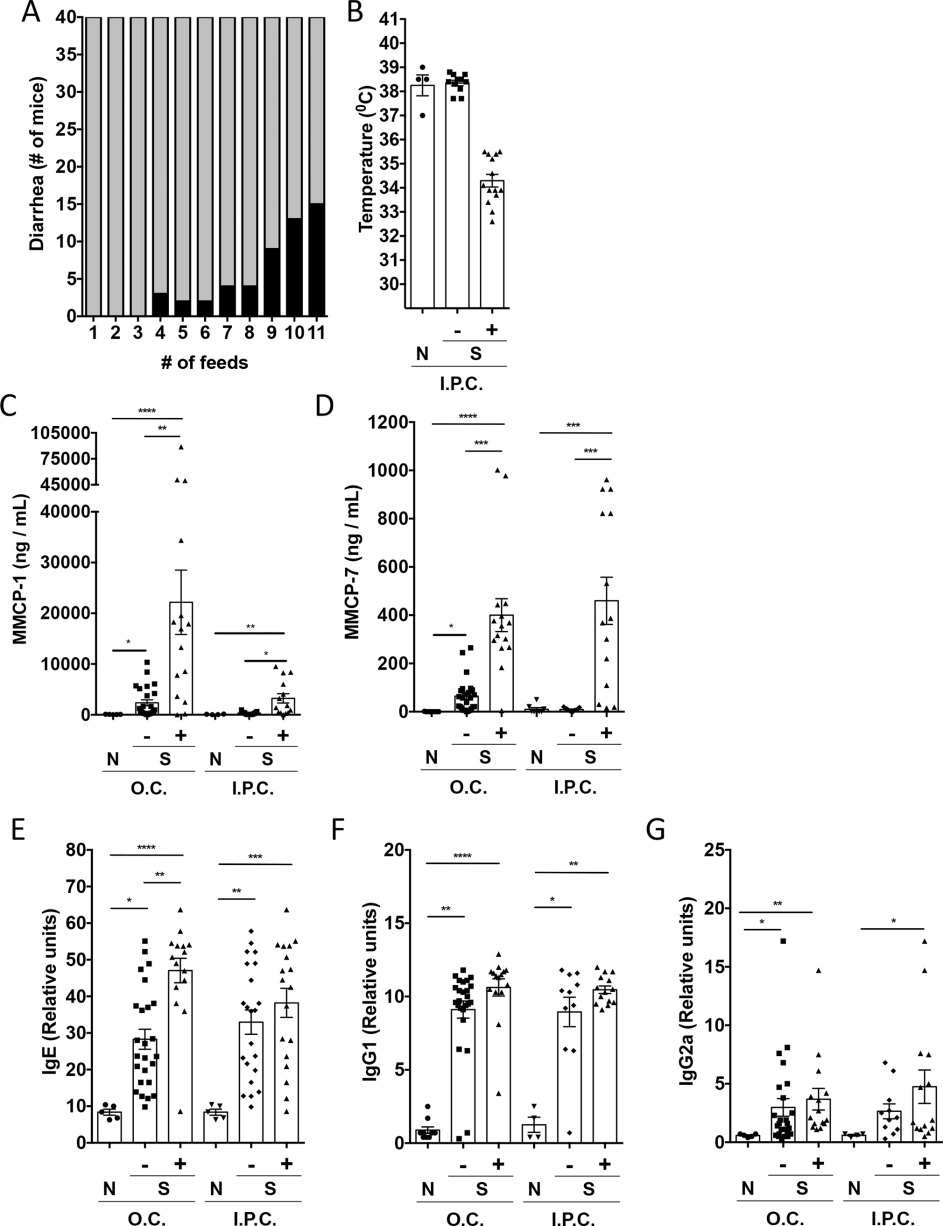
Contribution of mast cell subsets to manifestations of food allergy in the allergic diarrhea model. Balb/c mice (n = 40) were sensitized and orally (O.C.) or intraperitoneally (I.P.C.) challenge with OVA to induce diarrhea. Controls (N) included naïve mice that were not sensitized but challenged with OVA. Number of Balb/c mice developing diarrhea (black bars) or being asymptomatic (gray bars) in the OVA sensitized group (S) after each oral antigen challenge (A). Body temperature was measured 30 minutes after I.P.C. (B). MMCP-1 (C), MMCP-7 (D), OVA-specific IgE (E), OVA-specific IgG1 (F) and OVA-specific IgG2a (G) serum levels were measured 30 minutes after O.C. or I.P.C. Horizontal bars represent the mean value; error bars represent SEM. *p<0.05, ** p < 0.01, ***p<0.001, ***p<0.0001.

We examined mast cell subset activation in a model of gastrointestinal food allergy (Fig 5). Fifteen of 40 mice developed acute diarrhea in response to oral OVA challenge (Fig 5A) and again responders and non-responders were examined among the sensitized group. Mice did not have a drop in body temperature after oral OVA challenge (not shown). Mice with gastrointestinal symptoms had higher levels of IgE (Fig 5E), MMCP-1 (Fig 5C, Table 5), as well as MMCP-7 levels (Fig 5D, Table 5). Plasma histamine and serotonin levels after oral challenge were not associated with symptoms in this model (not shown). Spearman`s coefficient indicated a positive correlation between MMCP-1 and MMCP-7, IgE, IgG1, and IgG2a (Table 6).

**Table 5:** Adjusted p values comparing MMCP-1 and MMCP-7 levels between naïve, non-responder (NR) and responder (R) Balb/c mice after oral or intraperitoneal challenge.

	Oral		Intraperitoneal	
	MMCP1	MMCP7	MMCP1	MMCP7
**Naïve vs NR**	0.0452	0.0037	0.6513	> 0.9999
**Naïve vs R**	< 0.0001	< 0.0001	0.0063	0.0003
**NR vs R**	0.0079	0.0006	0.0331	0.0005

**Table 6:** Correlation between mast cell mediators measured in serum and diarrhea in Balb/c mice after oral challenge.

Oral challenge Balb/c (Diarrhea)							
	Diarrhea +/-	MMCP-1	MMCP-7	HISTAMINE	SEROTONIN	IgE	IgG1
**MMCP-1**	**0.660**	** **	** **	** **	** **	** **	** **
	(0.000)						
**MMCP-7**	**0.722**	**0.883**	** **	** **	** **	** **	** **
	(0.000)	(0.000)					
**HISTAMINE**	**-0.438**	**-0.512**	**-0.465**	** **	** **	** **	** **
	(0.041)	(0.015)	(0.029)				
**SEROTONIN**	**-0.078**	**-0.315**	**-0.136**	**0.316**	** **	** **	** **
	(0.716)	(0.134)	(0.525)	(0.215)			
**IgE**	**0.555**	**0.806**	**0.848**	**-0.145**	**0.035**		** **
	(0.000)	(0.000)	(0.000)	(0.519)	(0.870)		
**IgG1**	**0.448**	**0.466**	**0.503**	**-0.516**	**-0.169**	**0.423**	
	(0.004)	(0.002)	(0.001)	(0.014)	(0.430)	(0.007)	
**IgG2a**	**0.246**	**0.340**	**0.411**	**-0.162**	**0.065**	**0.530**	**0.302**
	(0.125)	(0.032)	(0.008)	(0.472)	(0.764)	(0.000)	(0.058)

Bolded values indicate spearman correlation coefficient, values in brackets indicate p value.

Systemic challenge of the mice did not result in diarrhea but instead systemic anaphylaxis with drop in core body temperature in a subset of mice (Fig 5B). Correlation analysis demonstrated a significant correlation of anaphylaxis severity (drop in body temperature) with MMCP-1 and MMCP-7, while histamine trended toward correlation (r = 0.486, p = 0.069) (Fig 5, Tables 5 and 7). Antibody levels did not correlate positively with anaphylaxis, and surprisingly, a negative correlation between IgE levels and drop in body temperature was observed. Histamine levels significantly correlated with MMCP-7but not MMCP-1 after intraperitoneal challenge of Balb/c mice.

**Table 7:** Correlation between mast cell mediators measured in serum and anaphylaxis symptoms in Balb/c mice after intraperitoneal challenge.

Intraperitoneal challenge Balb/c (Anaphylaxis)							
	Drop T	MMCP-1	MMCP-7	HISTAMINE	SEROTONIN	IgE	IgG1
**MMCP-1**	**0.465**						
	(0.019)						
**MMCP-7**	**0.763**	**0.617**					
	(0.000)	(0.001)					
**HISTAMINE**	**0.386**	**0.277**	**0.466**				
	(0.069)	(0.200)	(0.025)				
**SEROTONIN**	**-0.298**	**0.190**	**-0.153**	**0.073**			
	(0.189)	(0.408)	(0.509)	(0.758)			
**IgE**	**-0.528**	**0.076**	**-0.341**	**-0.344**	**0.330**		
	(0.011)	(0.736)	(0.120)	(0.126)	(0.182)		
**IgG1**	**-0.093**	**0.476**	**0.148**	**-0.064**	**0.075**	**0.434**	
	(0.658)	(0.016)	(0.479)	(0.773)	(0.745)	(0.044)	
**IgG2a**	**0.055**	**0.195**	**0.172**	**0.078**	**-0.174**	**0.469**	**0.322**
	(0.796)	(0.351)	(0.411)	(0.725)	(0.450)	(0.027)	(0.117)

Bolded values indicate spearman correlation coefficient, values in brackets indicate p value. Drop T = drop in body temperature.

## Discussion

Mast cells are the main effector cells in IgE-mediated allergic diseases [1], including food allergy [20, 21], and represent the major source of mediators that contribute to clinical manifestations of food allergy [22]. During the allergic response, allergen cross-links IgE bound to MCs via FcεRI, resulting in activation and release of preformed granule contents, rapidly synthesized lipid mediators or cytokines and chemokines. Although this is the central mechanism in food allergy, the outcome of exposure to foods can be highly variable in terms of both type of manifestations and severity. MCs are a highly heterogeneous population of cells, as recently reviewed by Cildir et al [23]. Tissue microenvironment regulates MC expression of proteases and functional specialization [8, 11]. We hypothesized that these different MC subsets would underlie different manifestations of food allergy. To test this, we evaluated mast cell subset activation in two different mouse models presenting with gastrointestinal or anaphylaxis symptoms in response to oral or systemic challenge.

We took advantage of responders and non-responders in the two models of food allergy to study the association of MMC and CTMC activation with symptoms rather than sensitization. Although MMCP-1 is a commonly used biomarker of hypersensitivity responses [24, 25], it surprisingly did not correlate with the outcome of systemic anaphylaxis and was elevated after oral challenge of sensitized mice independent of clinical outcome. Anaphylactic symptoms were observed in mice with evidence of CTMC activation as identified by MMCP-7 release into the serum. This is consistent with the findings of Strait et al., [26] that showed that allergen must be absorbed to induce systemic symptoms (in contrast to triggering reactions in the gastrointestinal tract that then are immunologically spread throughout the organism). Therefore, approaches to limit allergen absorption may be effective in suppressing systemic reactions to foods, as is supported by our previous findings showing that heated milk and egg allergens, that are not absorbed across the intestinal epithelium intact, cannot trigger anaphylaxis by the oral route in mice [27, 28]. We do not hypothesize that MMCP-7 is directly associated with the pathophysiology of systemic anaphylaxis, which is mediated by histamine and platelet activating factor [5]. CTMCs have been reported to produce substantially more histamine than MMCs [29, 30], suggesting why MMCP-7 may be more closely associated with systemic anaphylaxis than MMCP-1.

Previous studies have demonstrated that intestinal MCs are required for experimental oral allergen-induced diarrhea [7]. Local mast cell activation releases mediators that act on intestinal epithelial cells to induce ion secretion and increase permeability, leading to diarrheal symptoms [31]. A key feature of the allergic diarrhea model, but not the anaphylaxis model, is intestinal mastocytosis. There are very few mature MCs at steady state in the intestine of mice housed in clean conditions, in contrast to skin which contains a robust population of MCs. A greater degree of clinically relevant mast cell heterogeneity was described by Chen et al [32], who identified a subset of agranular MMCs that produced large amounts of the cytokine IL-9 and that were a key intermediate between the generation of allergen-specific Th2 cells during sensitization and intestinal mastocytosis required for allergic symptoms. These “MMC9” cells expressed and secreted MMCP-1 and would not be distinguished by the markers used here. Gastrointestinal manifestations of food allergy have been described to be dependent on serotonin [7]. We did not observe a correlation of peripheral serotonin levels with diarrhea symptoms. This does not rule out a role for serotonin in symptoms. While the major source of serotonin in the periphery is derived from enterochromaffin cells in the gastrointestinal tract, neuronal serotonin may be an important mediator of symptoms without leading to enhanced peripheral serotonin levels.

Interestingly we also observed an association of diarrhea symptoms with MMCP-7, despite the lack of evidence of systemic anaphylaxis in these mice. This may be secondary to the increased permeability induced by gastrointestinal MC activation [33], but it is still unclear why connective tissue mast cell activation was not associated with systemic anaphylaxis as measured by drop in body temperature in this model system. Others have reported co-existing symptoms of diarrhea and systemic anaphylaxis [17, 18], and method and route of sensitization may play a role in determining presence or absence of systemic symptoms. We did not assess objective parameters of systemic anaphylaxis other than body temperature, and perhaps other measures such as vascular leak would have revealed evidence of systemic anaphylaxis.

Although we focused on responders and non-responders within each strain and model, a number of important strain differences have been reported that contribute to disease manifestations. There have been differing reports on the role of TLR4 in susceptibility to food-induced anaphylaxis [16, 34], but we have found that C3H mice are susceptible to food-induced anaphylaxis independent of TLR4 mutations [16]. C3H/HeJ mice express an isoform of mRasGRP4, a MC-restricted protein, that makes them hyporesponsive to stimulation with PMA [35]. Mice lacking RasGRP4 have normal MC development and slightly impaired IgE-mediated activation [36], indicating that RasGRP4 is unlikely to explain susceptibility to food-induced anaphylaxis. C3H/HeJ mice also harbor a mutation in DOCK8 [37]. Furthermore, DOCK8 deficiency in humans is associated with clinical allergy to foods, implicating this as a key susceptibility gene to food-induced anaphylaxis [38]. The mechanism linking DOCK8 deficiency to systemic anaphylaxis remains to be defined. Although the genetic basis of susceptibility to gastrointestinal manifestations has not been identified, strain-dependent IL-9 expression in the gastrointestinal tract that drives an increase in tissue mast cells is closely associated with disease development [32].

Gastrointestinal manifestations of food allergy were more highly correlated with serum allergen-specific IgE levels than systemic anaphylaxis, even anaphylaxis elicited by oral challenge. This lack of correlation of IgE and anaphylaxis could be due to sampling of serum after allergen challenge. Systemic allergen could potentially suppress the detection of allergen-specific IgE. However, the lack of correlation of symptom severity with specific IgE levels is consistent with observations in human food allergy. Clearly additional factors, such as affinity of allergen binding and interaction of IgE with mast cells are important factors contributing to clinical reactivity. Although we did not directly test the IgE-dependence of the model systems, others have shown the central role of IgE in the local and systemic response to oral allergen challenge [7, 17]. However, non-IgE-mediated pathways have also been reported in some models of food allergy [39] or anaphylaxis [40, 41].

A common feature of both models was the increased MMCP-8 expression in the lungs of sensitized mice compared to naïve mice. MMCP-8 is unique to basophils [19]. We did not assess airway responses to challenge in either of these models, but it is intriguing that basophils are ideally situated to contribute to respiratory symptoms. Further studies are needed to address this point.

Strengths of the study include the use of epicutaneous sensitization which is a clinically relevant route of allergen exposure, a clinically relevant adjuvant (staphylococcal enterotoxin B), and a clinically relevant allergen (ovalbumin from egg). Peanut is another highly relevant food allergen, but is a poor inducer of reactions by oral challenge in our experience. Another strength is the use of responders and non-responders to identify immune activation that is clinically meaningful within the mouse model. Limitations of the study include the use of associations with MC subset activation rather that targeted depletion of subsets. However, the strain-dependence of the various models makes the use of genetic tools burdensome. Our results pointing to the clinical importance of mast cell heterogeneity call for similar studies performed in human cohorts. Performing serum profiling on food allergic cohorts undergoing supervised oral food challenge would assist in identifying the contribution of mast cell subsets to different manifestations of food allergy. In particular, identifying pathways responsible for systemic reactions may identify biomarkers of patients at risk, and identify candidates for therapeutic targeting.

## Supporting information

S1 File Excel file providing Ct values for Mcpt genes as shown in Fig 2. (XLSX)

S2 File Excel file providing data on C3H/HeJ mice. Body temperature, MMCP-1, MMCP-7, Histamine, IgE, IgG1, IgG2a, Serotonin, Leukotrienes.
(XLSX)

S3 File Excel file providing data on Balb/c mice. Symptoms, body temperature, MMCP-1, MMCP-7, Histamine, IgE, IgG1, IgG2a, Serotonin, Leukotrienes.
(XLSX)
